# Office Visitor

**Published:** 1906-01

**Authors:** 


### OFFICE VISITOR.

She brushed past the office girl and in a loud, high pitched voice exclaimed, "Yes, I'll just go right in to the Doctor's room. Why, good morning, Doctor! Some of your work has failed. I thought your work never did come out, but it did. This filling (pointing to the tooth and trying to talk rapidly with her mouth wide open) came out about a month or two ago, no, what am I saying! it was last summer, when I was in the mountains. You know, there was the nicest kind of a young dentist there in the hotel, and I got him to look at it, and he said you ought to have filled it with gold and it would have stayed there, and he wanted to charge me two dollars to fix it right. But I told him just to hush, he couldn't run down my old dentist, and he laughed and I laughed and we had lots of fun over it. Hee! hee! hee! and I told him I was afraid he would hurt me, and that I would make a date with him later when I felt better and not so nervous. I wasn't going to let him work on my mouth; I was just coming back to you and make you do it over for nothing, right. Hee! Hee! Hee! But, you know, I reckon I ought to pay you something for doing it over—if it was your fault—for it caused that little ninnie of a dentist to pay me more attention than a little. He took me to ride and to the grand ball, and danced with me every day and set me up to ice-cream and coca-cola and I don't know what all. Oh! it was funny. Hee! Hee! Hee! He said there was lots more to be done to my teeth and I guess he was working to get the job. Hee! Hee! Hee! But he never got it. No, sir! I was afraid he would try to get even with me and I knew you would fix this one over for nothing and do the others very reasonable. Now, won't you, dear Doctor?" After explaining the cause of the failure, we fixed her up satisfactorily and she departed, with a hearty goodby and giggling.

